# Influenza A (H1N1): outbreak management in a dialysis unit and clinical outcomes of infection in chronic hemodialysis patients

**DOI:** 10.1590/2175-8239-JBN-2019-0180

**Published:** 2020-03-23

**Authors:** Carlucci Gualberto Ventura, Felício Lopes Roque, Itanilton Queiroz de Sousa, Renata Desordi Lobo, Claudio Luders

**Affiliations:** 1 Hospital Sírio-Libanês, Centro de Nefrologia e Diálise, São Paulo, SP, Brasil.; 2 Hospital Sírio-Libanês, Controle de Doenças Infecciosas, São Paulo, SP, Brasil.

**Keywords:** Renal Dialysis, Influenza A virus, Disease Outbreaks, Hemodialysis Unit, Hospital, Diálise Renal, Vírus da Influenza A, Surtos de Doenças, Unidades Hospitalares de Hemodiálise

## Abstract

**Introduction:**

Chronic hemodialysis (HD) patients are considered to be at high risk for infection. Here, we describe the clinical outcomes of chronic HD patients with influenza A (H1N1) infection and the strategies adopted to control an outbreak of influenza A in a dialysis unit.

**Methods:**

Among a total of 62 chronic HD patients, H1N1 infection was identified in 12 (19.4%). Of the 32 staff members, four (12.5%) were found to be infected with the H1N1 virus. Outcomes included symptoms at presentation, comorbidities, occurrence of hypoxemia, hospital admission, and clinical evaluation. Infection was confirmed by real-time reverse transcriptase polymerase chain reaction.

**Results:**

The 12 patients who had H1N1 infection did not differ significantly from the other 50 non-infected patients with respect to age, sex, dialysis vintage, dialysis modality, or proportion of comorbidities. Obesity was higher in the H1N1-infected group (41.5 vs. 4%, p<0.002). The most common symptoms were fever (92%), cough (92%), and rhinorrhea (83%). Early empirical antiviral treatment with oseltamivir was started in symptomatic patients and infection control measures, including the intensification of contact-reduction measures by the staff members, antiviral chemoprophylaxis to asymptomatic patients undergoing HD in the same shift of infected patients, and dismiss of staff members suspected of being infected, were implemented to control the spread of infection in the dialysis unit.

**Conclusion:**

The clinical course of infection with H1N1 in our patients was favorable. None of the patients developed severe disease and the strategies adopted to control the outbreak were successful.

## RESUMO

**Introdução:**

Pacientes em hemodiálise (HD) crônica apresentam risco elevado para infecções. O presente estudo descreve os desfechos clínicos de pacientes em HD crônica com infecção pelo vírus influenza A (H1N1) e as estratégias adotadas para controlar um surto de influenza A numa unidade de diálise.

**Métodos:**

Doze (19,4%) de 62 pacientes em HD crônica e quatro (12,5%) de 32 funcionários desta unidade de diálise apresentaram infecção pelo vírus H1N1. Os desfechos incluíram sintomas à apresentação, comorbidades, ocorrência de hipoxemia, internação hospitalar e avaliação clínica. A presença de infecção foi confirmada por reação em cadeia da polimerase via transcriptase reversa (RT-PCR) em tempo real.

**Resultados:**

Os 12 pacientes com infecção por H1N1 não diferiram significativamente dos 50 pacientes sem infecção no tocante a idade, sexo, tempo em diálise, modalidade de diálise e percentual de comorbidades. O percentual de obesidade foi mais elevado no grupo com infecção por H1N1 (41,5% vs. 4%, p<0,002). Os sintomas mais comuns foram febre (92%), tosse (92%) e rinorreia (83%). Os pacientes foram submetidos a tratamento antiviral com oseltamivir e medidas de controle (intensificação das medidas de redução de contato pelos funcionários da clínica, quimioprofilaxia com antiviral para pacientes assintomáticos em HD na mesma sala dos pacientes com infecção e afastamento de funcionários da clínica com suspeita de infecção) para controlar a disseminação da infecção pela unidade de diálise.

**Conclusão:**

O curso clínico da infecção por H1N1 em nossos pacientes foi favorável. Nenhum evoluiu para doença grave e as estratégias adotadas foram efetivas no controle do surto.

## INTRODUCTION

Influenza A (H1N1) is associated high morbidity and mortality, particularly among elderly people, pregnant women, and patients with chronic diseases^1^. Patients with end-stage renal disease (ESRD) are considered to be at high risk for H1N1 infection because of their altered immune status. Immune dysfunction has complex and multifactorial causes including defects in complement activation, neutrophil function, B-cell and T-cell function^2^^,^ 3. An additional risk factor is undergoing hemodialysis (HD) in concurrent sessions, which implies exposure to other patients and health care personnel, thus facilitating cross-contamination within the dialysis unit. In addition, prolonged viral shedding in immunosuppressed individuals can increase the risk of viral transmission. One study demonstrated that the rates of H1N1 influenza were significantly higher among patients on HD than among those on peritoneal dialysis^4^. Moreover, the treatment of patients with influenza has a significant impact on outpatient services^5^.

The clinical presentation of H1N1 infection can range from mild respiratory symptoms to acute respiratory insufficiency, and HD patients tend to present a greater number of clinical complications and to develop severe or life-threatening disease. Underlying chronic medical conditions such as diabetes mellitus, heart disease, and lung disease contribute to the high risk of influenza complications among HD patients^6^.

Influenza outbreaks are usually seasonal, peaking during the winter in the southern hemisphere. Although most of Brazil’s territory lies in the tropics, more than 60% of the population, including from São Paulo state, lives in the subtropical area with mild to cold winters. The cases of infection by H1N1 have been reported with an increasing frequency between the months of June and September. Influenza vaccination campaigns have been conducted annually, between the end of April and May. Compared to previous years, seasonal influenza activity commenced early in São Paulo during the 2016 year, before the vaccination campaign period. In March 2016, a high number of cases of acute respiratory infection caused by H1N1 virus were reported in the city of São Paulo. At that time, we also witnessed an outbreak of H1N1 in our dialysis unit (outpatient setting). This study describes the clinical evolution of individuals infected with the H1N1 virus and discuss the management strategies that should be adopted to control an influenza outbreak in a dialysis unit.

## MATERIAL AND METHODS

Between March and April 2016, we evaluated 16 individuals infected with the H1N1 virus -12 adult patients with ESRD receiving regular HD therapy and four staff members- during a H1N1 outbreak at the dialysis unit of Sírio-Libanês Hospital, a private hospital in the city of São Paulo. At that time, the unit had 15 dialysis stations and served a total of 62 patients. The dialysis unit staff comprised six physicians, four nurses, 18 nursing technicians, one nutritionist, and three receptionists. Patients were on conventional HD (3 to 4 hours per session, three times a week) or intensified HD (nocturnal HD: 8 hours per session, three times a week or daily HD: 2-2.5 hours per session, five to six times a week). Our dialysis unit did not have patients on peritoneal dialysis.

Patients and staff who had one or more symptoms resembling those of seasonal influenza, including fever (>37.4°C), cough, rhinorrhea or nasal congestion, body aches, sore throat, and headache were investigated for influenza.

The diagnosis of H1N1 infection was confirmed by real-time RT-PCR (rRT-PCR) test using nasopharyngeal swab specimens, in accordance with guidelines from the Centers for Disease Control and Prevention^7^. For all patients, the diagnosis and decisions regarding treatment, including the choice of antiviral therapy to be administered, were standardized and were made by the attending physician. Demographic and clinical data were obtained from medical records. The following information was reviewed: symptoms at presentation, comorbidities, HD modality, occurrence of hypoxemia, laboratorial parameters, hospital admission, and clinical outcomes.

During the outbreak, four staff members developed flu-like symptoms and were diagnosed with influenza A. They were temporarily dismissed from the dialysis unit and were duly treated. This study was approved by the Research Ethics Committee of Sírio-Libanês Hospital (HSL 2017-74). Patient anonymity was guaranteed, and the requirement to obtain informed consent was waived because of the retrospective nature of the study.

### INFECTION CONTROL MEASURES

After the first case of H1N1 infection had been confirmed, the following strategies were implemented to control the spread of infection in the dialysis unit: all dialysis patients were required to wear a surgical mask before entering the dialysis room. The health care staff intensified the use of contact-reduction measures and standard precautions, including the use of surgical masks, non-sterile gloves, and hand hygiene with soap and water or alcohol-based hand sanitizer. Early empirical antiviral treatment with oseltamivir was started in the patients and staff members who were suspected of being infected. Antiviral chemoprophylaxis was offered to asymptomatic patients undergoing HD in the same shift of infected patients. All patients and staff members were immediately vaccinated with the 2016 influenza vaccine, regardless of their vaccination status. The staff members suspected of being infected were removed from the dialysis unit for a 7-day period. Precautionary measures were maintained for 7 days after symptom onset or for at least 24 h after the symptoms had resolved, whichever was longer.

### ANTIVIRAL THERAPY AND CHEMOPROPHYLAXIS

The H1N1-infected patients were treated with an initial oral dose of 75 mg of oseltamivir followed by 30 mg after each HD session. Patients on daily dialysis received oseltamivir daily after HD session. The treatment was initiated as soon as possible after the onset of symptoms (if necessary, even before infection had been confirmed by real-time RT-PCR). Antiviral chemoprophylaxis with oseltamivir (30 mg) was administered after each HD session for a 7-day period. Staff members infected with H1N1 were also treated with oseltamivir for a 5-day period.

### VACCINATION

Seasonal influenza vaccination has been provided free of charge to all patients on dialysis and health-care professionals. Administration of the seasonal vaccine 2015-2016 (tetravalent vaccine with components of A/California/7/2009 (H1N1) pdm09; influenza A/Hong Kong/4801/2014 (H3N2); B/Brisbane/60/2008; and B/Phuket/3073/2013) was performed in a single dose, between April 2 and May 22, 2016. Seasonal vaccine formulation in the 2014-2015 campaign contained three strains: A/California/7/2009 (H1N1) pdm09, A/Texas/50/2012, B/Massachusetts/2/2012 and was also administered in a single dose. Vaccination status of 2014-2015 campaign was determined directly from the patients with interview and from local dialysis unit records during the outbreak.

### STATISTICAL ANALYSIS

Continuous variables are reported as mean (± SD) or median (interquartile range), as appropriate, whereas categorical variables are presented as frequency (%). Differences in variable distributions between two groups were analyzed using the nonparametric Mann-Whitney U test (continuous variables), or Fisher’s exact test (categorical variables), as the distributions of all variables were not normal. All statistical analyses were performed with SPSS Statistics software package (version 18.0, IBM Corporation, USA). Two-sided p-values <0.05 were considered statistically significant.

## RESULTS

Among a total of 62 chronic HD patients in our dialysis unit, H1N1 infection was identified in 12 (19.4%). Of the 32 staff members working in the dialysis unit, four (12.5%) were found to be infected with the H1N1 virus.

Table 1 shows the demographic and clinical characteristics of the patients. The 12 patients who had H1N1 infection did not differ significantly from the other 50 non-infected patients with respect to median age, sex, mean time on dialysis, or dialysis modality. The proportion of comorbidities was similar, except for obesity (body mass index >30 kg/m^2^), that was higher in the H1N1-infected group than in the non-infected group (41.5 vs. 4%, p<0.002). Although five H1N1-infected patients (41.5%) were obese, none were morbidly obese.

**Table 1:** Demographic and clinical characteristics of hemodialysis patients with or without influenza A (H1N1) infection.

	Infected patients	Non-infected patients	p-value
	(N=12)	(N=50)	
Age (years); median (range)	68.5 (58–78.7)	70 (57.5–83.5)	0.82
Male gender	10 (83.3)	36 (72)	0.71
HD vintage (months)	27.3 ± 24.5	36.9 ± 30.4	0.29
Dialysis modality			0.30
Conventional	10 (83.3)	32 (64)	
Intensified	2 (16.7)	18 (36)	
Comorbidities			
Diabetes mellitus	7 (58.3)	19 (38)	0.33
Obesity	5 (41.6)	2 (4)	0.002
Heart disease	7 (58.3)	22 (44)	0.52
Chronic lung disease	1(8.3)	4 (8)	0.99
Malignancy	2 (16.7)	2 (4)	0.17
Immunosuppression	2 (16.7)	5 (10)	0.61
Leukocytes (10^3^/mm^3^)	6.7 ± 2.2	6.6 ± 2.1	0.85
Lymphocytes (10^3^/mm^3^)	1.0 ± 0.3	1.4 ± 0.4	0.007
CRP (mg/dL)	2.8 ± 3.0	0.6 ± 0.6	0.029
Serum albumin (g/dL)	3.7 ± 0.3	3.9 ± 0.3	0.029
Vaccinated in previous year	9 (75)	38 (76)	NS
(2014-2015 vaccine)			
Duration of antiviral therapy (days)	5 ± 2	-	-
Hospital rate	3 (25)	-	-
Length of hospital stay (days)	4 ± 0	-	-

Continuous variables are reported as mean (±SD). Categorical variable are reported as number (%). NS: non-significant

Among the HD-infected patients, symptom onset occurred between March 21 and April 11, 2016. The presenting symptoms are summarized in Table 2. The most common symptoms were cough and fever. Of the 12 patients, eleven (92%) had low-grade fever and, one patient (8%) was afebrile. Gastrointestinal symptoms (diarrhea, nausea, and vomiting) were observed in only one patient. The estimated median time from symptom onset to initiation of antiviral treatment was 2 days. The diagnosis of H1N1 infection was confirmed by real-time RT-PCR of nasopharyngeal swab samples in ten of the 12 symptomatic patients (two patients refused sample collection). All but one of the patients received empiric antiviral treatment within the first 48 h after symptom onset, before the test results were available. The duration of treatment with oseltamivir was 5 days, except one patient who was treated for seven days at medical discretion. None of the patients experienced adverse events related to the use of oseltamivir. The total white blood cells count was similar in the two groups. The infected patients had C-reactive protein (CRP) significantly higher while the lymphocytes count and serum albumin were significantly lower compared with non-infected patients. The influenza vaccination rate (2015) was similar between infected patients (75%) and non-infected patients (76%).

**Table 2:** Clinical features on presentation of H1N1 infection in hemodialysis patients (N = 12).

Symptom	n (%)
Cough	11 (92)
Fever	11 (92)
Rhinorrhea	10 (83)
Myalgia	6 (50)
Bronchospasm	2 (17)
Sore throat	2 (17)
Dyspnea	2 (17)
Headache	2 (17)
Chills	1 (8)
Diarrhea	1 (8)
Oxygen saturation rate, mean (SD)	94 ± 2

Of the 12 H1N1-infected patients, three (25%) were hospitalized, each for a period of 4 days. The reasons for hospitalization were: exacerbation of chronic asthma; severe gastrointestinal symptoms, and an elderly patient with COPD. At admission, all patients underwent a computed tomography scan of the chest, which showed no evidence of pulmonary infiltrate in all cases. Neither of the hospitalized patients presented hypoxemia or required admission to the intensive care unit (ICU). Complete recovery was observed in all H1N1-infected patients.

**Figure 1:**
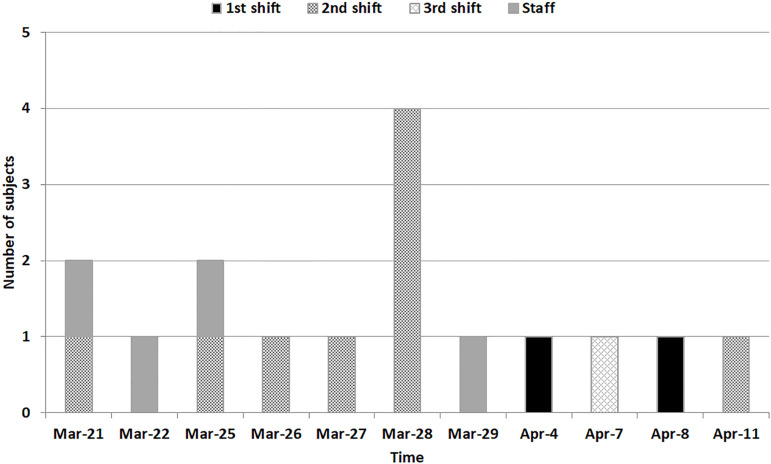
Distribution of H1N1-infected patients and staff members by dialysis shift.

The number of individuals infected in the period of outbreak and their distribution among the dialysis shifts are presented in Figure 1. The first case of the influenza A outbreak in our dialysis unit was admitted on March 21, 2016. That patient was placed under respiratory precautions as soon as the influenza symptoms were known to the staff physician. The second confirmed case was in a staff member (a receptionist) who was removed from the dialysis unit as soon as the infection was suspected. On March 25, infection was confirmed in a second patient who was on the same dialysis schedule (second shift) as the first infected patient, constituting an outbreak in the unit and leading to prompt implementation of infection control measures. Other six infected patients were identified between March 26 and 28. Of the 12 H1N1-infected patients, ten (83%) occurred in the same dialysis room: nine (75%), including the first case, during the second HD shift, and one in a subsequent HD shift (third shift). The two remaining patients were on the morning HD schedule (first shift). The analysis of the clinical background of the second shift patients was not significantly different compared to those patients on HD in the other shifts (Table 3).

**Table 3:** Demographic and clinical characteristics of hemodialysis patients in 2nd shift or other shifts.

	2nd Shift	Other Shifts	p-value
	N=15	N=47	
Age (years) median (range)	71 (58-89)	70 (55-80.5)	0.32
Male gender, *n* (%)	11 (73.3)	35 (74.4)	0.99
Comorbidities, *n* (%)			
Diabetes mellitus	4 (26.6)	22 (46.8)	0.23
Obesity	1 (6.6)	6 (12.7)	0.67
Heart disease	7 (46.6)	22 (46.8)	0.99
Chronic lung disease	2 (33.3)	3 (6.4)	0.58
Malignancy	2 (13.3)	2 (4.2)	0.25
Immunosuppression	2 (13.3)	5 (10.6)	0.99

The four H1N1-infected staff members were one receptionist, one nurse, and two nurse technicians. Infection with the H1N1 virus was confirmed by real-time RT-PCR in only three (75.0%); no samples for PCR was collected from one staff member. All of the infected individuals received the recommended treatment with oseltamivir.

The 2016 seasonal influenza vaccine was offered in advance to all patients under treatment at the dialysis unit during the outbreak. All staff members were required to be vaccinated.

## DISCUSSION

In this study, we have described the clinical course of documented H1N1 infection in 12 HD patients and the infection control measures implemented during an outbreak in a dialysis unit. There is a paucity of data regarding H1N1 infection in HD patients. Although case series related to the 2009 H1N1 pandemic have reported a relatively severe clinical course of infection in the dialysis population^8^^-^ 9, we observed an apparently milder evolution. None of our patients presented severe disease, developed hypoxemia, or required ICU admission, and all recovered completely.

The real-time RT-PCR used in our study is currently the most sensitive and specific diagnostic test for influenza, results becoming available 4-6 h after specimen submission. In our analysis, two (16%) of the patients with flu-like symptoms presented nasopharyngeal swab samples that tested negative by real-time RT-PCR. However, these two patients were considered infected based on their clinical symptoms and on the fact that those symptoms appeared during an influenza outbreak. In another study, 19% of patients with detectable H1N1 viral RNA in bronchoscopy samples had previously tested negative for the virus in upper respiratory tract samples^10^. Therefore, negative results in single respiratory specimens do not rule out H1N1 infection and the collection of multiple respiratory specimens is recommended when the level of clinical suspicion is high. The two patients who refused to have nasopharyngeal swab samples collected to test RT-PCR were both febrile and presented with the appropriate clinical syndrome and epidemiologic exposure.

In the present study, the hospital admission rate was 25%, higher than the 1-7% estimated for the general population of H1N1-infected individuals^11^. In a previous multicenter study involving 306 chronic dialysis patients infected with the 2009 H1N1 virus, a hospital admission rate of 34% was reported^9^. In our study, the hospital admissions were due to complications of comorbid medical conditions. Two hospitalized patients had diabetes and cardiovascular disease and one had chronic obstructive pulmonary disease. Patients with chronic kidney disease are at a higher risk for hospitalization as a consequence of the comorbid conditions frequently associated with the primary renal disease^12^^,^ 13.

In the above mentioned multicenter study, the authors reported that the incidence of pneumonia was 22.5%^9^, whereas we observed no case of pneumonia in our analysis. Similarly, two previous case series of H1N1-infected chronic HD patients in China^8^ and Korea^4^ reported the incidence of pneumonia to be 100% and 17%, respectively. These discrepancies might be partly attributable to differences among the studies in terms of the characteristics of study populations, as well as the fact that the above studies refer to the first year of H1N1 infection with less immunized population. The low incidence of complications in our study can be explained by the high rate of patients previously vaccinated 78.3% received the 2014-2015 vaccine), early diagnosis, and prompt antiviral treatment.

In all but one of the patients in our study population, antiviral treatment was started before the laboratory test results had been made available. Decisions regarding antiviral treatment should not await laboratory confirmation, and patients presenting with progressive illness should be treated empirically as soon as possible. In the general population of H1N1-infected individuals, early treatment with oseltamivir can reduce the duration of hospitalization and the risk of progression to severe disease requiring ICU admission or resulting in death^14^.

The dose of oseltamivir used for treatment and prophylaxis in our center was directed by the recommended dose for patients with ESRD receiving HD^15^. Although the ideal regimen for treating influenza in these patients is uncertain, pharmacokinetics studies showed that a 30 mg dose of oseltamivir given after HD sessions, provides sufficient exposure to allow safe and effective anti-influenza treatment and prophylaxis^16^^,^ 17.

Vaccination continues to be the best way to prevent influenza illness. However, previous studies have reported reduced vaccine efficacy in HD patients. Crespo et al, showed that a single dose of the influenza A H1N1/2009 vaccine was associated with a low serum-conversion rate of 33% in this population^18^. Despite the controversy regarding the efficacy of influenza vaccine among patients on dialysis^19^^-^ 20, a 2-year analysis of Medicare claims for dialysis patients showed that vaccination significantly reduced the risk of all-cause hospitalizations and deaths associated with influenza^21^.

In addition, we administered chemoprophylaxis with oseltamivir to all patients undergoing HD in the same room of infected patients to contain the spread of the virus. Chemoprophylaxis has been recommended to control influenza in populations at high risk of complications and where vaccine efficacy is reduced, particularly across individual outbreaks^22^. Chemoprophylaxis with antiviral provides an immediate and immune-independent intervention to prevent seasonal influenza-related illness. We did not offer chemoprophylaxis to the staff member once all of them were previously vaccinated and were removed from dialysis as soon as they presented any flu symptom.

The mechanisms of person-to-person transmission of H1N1 virus appear to be similar to those of seasonal influenza^6^, but we were unable to determine the relative contributions of each transmission route (droplet, aerosol, or contact) in our patients. Epidemiologic analyses support patient-to-patient transmission, however, this does not rule out viral transmission from infected health care personal. The four staff members diagnosed with H1N1 had contact with patients undergoing HD in the second shift. In addition to standardized data collection, viral RNA sequencing should be performed when possible to enable the understanding of transmission events^23^.

It is an important fact that the majority of influenza infections were spreading in the same room. A risk factor for the spread of viruses in dialysis units is the practice of HD in concurrent sessions in a single space, which is inherently associated with exposure to other HD patients and health care staff^5^. In our dialysis center, we have one dialysis room specific for isolating patients with transmissible diseases, nevertheless, it is not feasible to isolate infected patients in other rooms during the HD sessions. The strength of this study remains in the fact that although patients were treated in the same dialysis room, the infectious outbreak was controlled successfully. After the infection-control interventions were implemented, the illness was diagnosed in four patients. The two cases restricted to the first shift had no contact with other infected patients, and is possible that the infection had come from the community.

Our study has several limitations. Because this was a retrospective analysis, we may have missed clinical information not documented. We also may have missed patients with asymptomatic infection because RT-PCR was collected only when patients were symptomatic. Chest radiograph was not available for all patients. However, all patients recovered completely and hypoxemia was not documented in any patient.

The control of the outbreak observed in the present study supports the use of the infection control measures implemented. In periods during which H1N1 virus circulates in a dialysis unit, it is likely that cases of flu-like illness represent H1N1 infection, and the initiation of antiviral therapy in suspected cases should not be delayed to wait for a definitive diagnosis based on laboratory tests. In such contexts, other infection control measures, such as the use of surgical masks by patients, their contacts, and dialysis unit staff members, together with antiviral chemoprophylaxis to asymptomatic patients undergoing hemodialysis in the same room, should be implemented and could help control the outbreak.

In conclusion, the clinical evolution of infection with the H1N1 virus was favorable. None of the patients developed severe disease, and the outbreak was successfully controlled.
